# Determining the Correlation Between Blood Loss and Clinical Findings Among Patients with Postpartum Hemorrhage

**DOI:** 10.1089/whr.2024.0103

**Published:** 2025-01-08

**Authors:** Rajani Dube, Subhranshu Sekhar Kar, Sanghamitra Satapathy, Biji Thomas George, Heena Garg

**Affiliations:** ^1^ Department of Obstetrics and Gynecology, RAK Medical and Health Sciences University, Ras Al Khaimah, UAE.; ^2^ Department of Pediatrics, RAK Medical and Health Sciences University, Ras Al Khaimah, UAE.; ^3^ Department of Obstetrics and Gynecology, Jagannath Hospital, Bhubaneswar, India.; ^4^ Department of General Surgery, RAK Medical and Health Sciences University, Ras Al Khaimah, UAE.; ^5^ Department of Obstetrics and Gynecology, Al Zahrawi Hospital, Ras Al Khaimah, UAE.

**Keywords:** blood loss, clinical findings, correlation, hypotension, postpartum hemorrhage, restlessness

## Abstract

**Background:**

There is a need for signs that will help the midwives or the health care providers attending deliveries to prevent the patient from going into hypovolemic shock, especially when immediate testing is not possible. The study aims to find the correlation between the clinical symptoms and blood loss in women with postpartum hemorrhage.

**Methods:**

It is a descriptive observational study conducted at the Department of Obstetrics and Gynecology, Maternity Hospitals. Women treated with either Misoprostol or Ergometrine during delivery were included in the study. Data were collected for Packed Cell Volume (PCV), Hemoglobin (Hb%), *etc.*; other investigations include general clinical condition, presence or absence of PPH, and amount of blood loss using laboratory reports.

**Results:**

The study has reported clinical findings and blood loss to identify the correlation between them. Only 4% of women suffered blood loss of more than 500 mL, *i.e.,* postpartum hemorrhage (PPH) occurred among them. The change in Hb% among the majority of the women was ranging between 0–0.5 gm% (71.5%). Most cases (72.72%) had tachycardia followed by palpitation (10.90%). Blood loss exceeding 1500 mL was correlated with hypotension, restlessness, and oliguria.

**Conclusions:**

Extra vigilance is needed to identify women at risk and facilitate early intervention and treatment of PPH.

## Introduction

Postpartum hemorrhage (PPH) is a medical emergency and description of an event, following delivery, and not a diagnosis. It is the consequence of excessive bleeding from the placental implantation site, trauma to the genital tract and adjacent structures, or both.^1^ Primary PPH is defined as the loss of 500 mL or more of blood in a woman during the first 24 hours after delivery of the baby or any loss (even <500 mL) that brings about hemodynamic changes in the mother. The quantity of blood loss during vaginal delivery has been reported varyingly in the literature depending on the population. It varies from 300 mL to 600 mL;^2,3^ however, the generally accepted quantity is 500 mL.^4^ In developing countries, the women have a smaller build, have less blood volume, are malnourished, and have a lower antenatal Hemoglobin (Hb%), which makes them develop hemodynamic instability with a lesser amount of blood loss. Therefore, in them, a lower value of blood loss is taken.^2^ Whereas, PPH after the first 24 hours is considered secondary PPH or late PPH. Excessive bleeding during the third stage of labor is, therefore, primary rather than secondary.

Physiological changes during pregnancy include red blood cells increased by 30% and blood volume increased by about 40%. However, this expansion in blood volume varies in certain conditions such as preeclampsia.^5^ Therefore, this blood loss could cause ischemic injury in one patient and can be compensated in another. PPH can be categorized as minor with a loss of 500–1000 mL, moderate with 1001–2000 mL blood loss, and severe with >2000 mL blood loss.^6^ PPH is a major cause of maternal mortality worldwide;^7^ however, its incidence rate is higher in developing or low-income countries.^8^ In accounts where mortality is prevented, PPH can still cause serious morbidities such as hysterectomy, coagulopathy, acute respiratory distress syndrome, hypovolemic shock, and infertility.^9^

According to a study, around 30% of postpartum blood loss is underestimated by health care providers.^10^ Therefore, robust detection of blood loss is required, and there is a need to record clinical symptoms. The body’s response to this blood loss is to activate the hemodynamic compensatory mechanism.^11^ Therefore, the body presents with clinical findings of weakness, restlessness, sweating, dizziness, palpitation, hypotension, and tachycardia.^11^ It is important to record these clinical symptoms, as they can be an indicator of hypovolemia in patients, and a speedy intervention could be provided without any delay. As the clinical findings are an important factor in detecting hypovolemia in patients, there is a need to analyze the relevance of these findings with the hemorrhage. This study aims to find the correlation between the clinical symptoms and blood loss in women with PPH.

There is currently a lack of studies correlating clinical factors due to blood loss caused by PPH and analyzing clinical presentation due to the body’s compensatory mechanism to hypovolemia in PPH. It will also help in identifying the signs that will help the midwives or the health care providers attending deliveries to prevent the patient from going into hypovolemic shock, especially when immediate testing is not possible.

## Materials and Methods

**Fig. 1.**
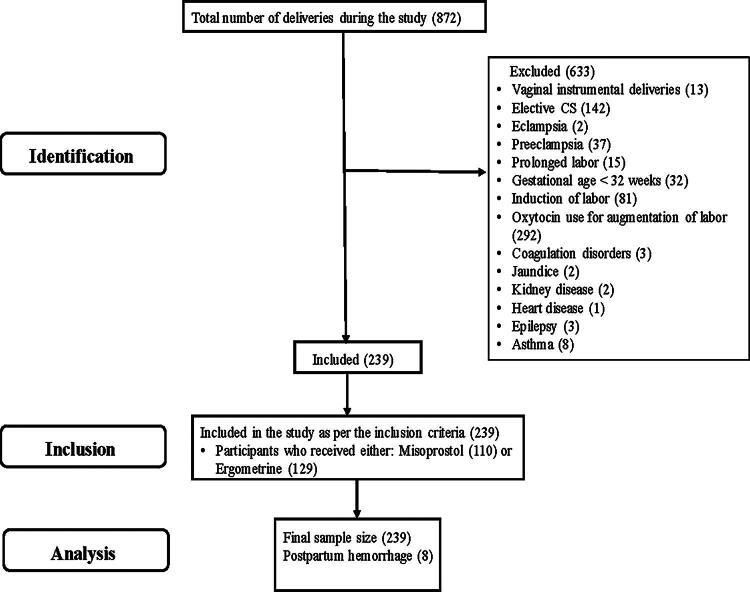
STROBE Flow Chart for Inclusion and Exclusion of Participants.

This observational study was conducted at the Department of Obstetrics and Gynecology of S.C.B. Medical College, Cuttack, and Jagannath Hospital, Bhubaneswar, in India. This study was approved by the Institutional Review Board (IRB) of SCB Medical College and Hospital (No.1024/2002). A sample size of 239 women was selected for the study who were given either Misoprostol (three oral tablets of 200 µg each) or Ergometrine (0.2 mg IV) for the prevention of PPH. All the participants had a normal vaginal delivery and therefore met the inclusion criteria. The exclusion criteria included those that had vaginal instrumental deliveries, elective CS, eclampsia, preeclampsia, prolonged labor, use of oxytocin for augmentation of labor, induction of labor, and gestational age <32 weeks. Other exclusion factors were the presence of diseases such as coagulation disorder, jaundice, kidney disease, heart disease, epilepsy, and asthma (Fig. 1). All the participants had given consent before participating in the studies. A Performa was prepared that included information such as lab reports of Packed Cell Volume (PCV), Hb%, other investigations, general clinical condition, presence or absence of PPH, and amount of blood loss. Vital signs were also recorded before the birth of the baby, every 15 minutes till 1 hour, every 30 minutes till 2 hours, and later at 4, 8, 12, 16, and 20 hours after delivery. Any potential side effects of the administered drugs were also reported. The immediate interventions were the blood transfusion if needed, administration of oxytocin if needed, manual removal of the placenta, and consequent evacuation of the uterus. Readings were taken from the blood collection drape to estimate the amount of blood loss after giving medical intervention. Also, clinical symptoms were noted along with taking lab values of PVC and Hb%.

The statistical analysis was done using the SPSS software version 25 to carry out the descriptive statistics, such as finding the percentage frequency of clinical signs and symptoms.

## Results

Table 1 presents the demographic details of 239 pregnant females, which shows that most of the women belong to the age group 25–29 years (36.4%) and 30–34 years (29.2%). Table 1 also shows that the majority of the women were primigravida (38.91%), followed by second gravida (20.0%). The majority of the women were nulliparous (48%), followed by primipara (27%) (Table 1).

**Table 1.** Demographic Details

Item	Measure	Frequency	Percentage
Age	15–19 yrs.	6	2.51
	20–24 yrs.	48	20.0
	25–29 yrs.	87	36.4
	30–34 yrs.	70	29.2
	35–39 yrs.	26	10.87
	>40 yrs.	2	0.83
Gravidity Distribution	Primigravida	93	38.91
	Second gravida	48	20.0
	Third gravida	33	13.8
	Fourth gravida	32	13.3
	Fifth gravida	12	5.02
	Sixth gravida	11	4.60
	Seventh gravida	4	1.67
	>Eighth gravida	6	2.51
Parity Status	0	96	40.1
	1	81	33.8
	2	39	16.3
	3	14	5.8
	4	4	1.7
	5	3	1.2
	>5	2	0.83

Table 2 shows that blood loss of 50–59 mL was noted in the majority of the cases (29.7%), followed by 100–149 mL (17.1%), 200–249 mL (16.7%), and 150–199 mL (14.2%). Table 2 also shows that 3.3% of women suffered blood loss of more than 500 mL *i.e.,* PPH occurred among them. The change in Hb% among the majority of the women was ranging between 0–0.5 gm% (59.8%) (Table 2).

**Table 2.** Total Amount of Blood Loss and Change in Hb Percentage

Items	Measures	Frequency	Percentage
Blood loss (mL)	0–49	4	1.7
	50–99	71	29.7
	100–149	41	17.1
	150–199	34	14.2
	200–249	40	16.7
	250–299	20	8.4
	300–349	10	4.1
	350–399	4	1.7
	400–449	4	1.7
	450–500	3	1.2
	>500	8	3.3
Change in Hb%	0–0.5	143	59.8
	0.6–1.0	86	35.9
	1.1–2	7	2.9
	2.1–5	2	0.83
	>5	1	0.41

The study reported that changes in clinical findings were seen among some of the women (*n* = 110) as a result of hemodynamic compensatory mechanisms in the body. Table 3 shows that most of the cases (72.72%) had tachycardia, followed by palpitation (10.90%). Dizziness was reported in 5.45% and sweating in 3.63% of the women. Weakness (3.63%), hypotension (2.72%), and restlessness (0.90%) exclusively occurred in a few of the women.

**Table 3.** Reported Clinical Findings at the End of the Third Stage

Condition	Frequency	Percentage
Tachycardia	80	72.72
Hypotension	3	2.72
Palpitation	12	10.90
Dizziness	6	5.45
Sweating	4	3.63
Restlessness	1	0.90
Weakness	4	3.63

The correlation analysis conducted in Table 4 shows that the majority of the women exhibited tachycardia as the manifestation of blood loss. Moreover, sweating was the main manifestation when the loss of blood was 500–1000 mL. Blood loss exceeding 1500 mL reported hypotension, restlessness, and oliguria. Lastly, confusion was not reported in any of the cases as a manifestation of PPH.

**Table 4.** Correlation of Amount of Blood Loss with Clinical Findings

Blood loss (mL)	Total	Individual	Findings
<500	231	111	Tachycardia
		6	Dizziness
		8	Palpitation
500<1000	5	4	Sweating
		3	Weakness
		1	Hypotension
1000–1500	2	2	Hypotension
		—	Confusion
>1500	1	1	Restlessness
		1	Oliguria
		0	Confusion

## Discussion

The findings show that 231 patients with blood loss (<500 mL) had tachycardia, dizziness, and palpitation. Patients with PPH, constituting only a small number of samples, presented symptoms such as sweating, weakness, hypotension, restlessness, and oliguria. The occurrence of PPH reported in this study was higher than that reported previously. At least 3.3% of a woman lost more than 500 mL, and five patients lost greater than 1000 mL.^12^ Similarly, the occurrence of PPH ranged from 7% in Oceania to 26% in Africa.^13^ The occurrence of severe PPH was lowest in Asia and highest in Africa. This high prevalence of PPH in this study might be because of the characteristics of the study population. This study has excluded multiple pregnancies and cesarean deliveries, as they correlated with the elevated risk of PPH.^14–16^ This study has analyzed singleton transvaginal deliveries.

The physiological modifications may hamper the initial identification of hypovolemia and delay treatment throughout postpartum and pregnancy. Similarly, no significant correlation was found by Hick et al. between hemoperitoneum and clinical signs.^17^ Furthermore, it is assumed that more than one-third of patients might be treated inadequately if surgical decisions were made based on clinical signs. Another study found similar data with no association between blood pressure and blood loss using an obstetric population in late pregnancy.^18^

Physiological changes in the cardiovascular system are even more significant within the postpartum period and late pregnancy. Some variables, in the case of PPH, have been recommended to enhance clinical evaluation for PPH treatment, such as visual estimation of blood loss, blood loss rate, and clinical signs and symptoms, but have not been adequately investigated. Scholars recommend modifying the blood-loss-based demonstration of PPH to a system of indications of hypovolemia. Previously, classes of hemorrhage-correlating indications were used to propose a hypovolemic shock classification system with the extent of blood loss and a fluid replacement process.^19–21^

Based on this classification, a blood loss of less than 1000 mL occurred with a compensated shock, and no modification or minimal change occurred in clinical signs. Significant changes can be seen after a blood loss of more than 1000 mL in heart rate and blood pressure. Hypotension would occur after a loss of 25%–35% of blood volume, and significant shock occurs after a 40% blood loss with significant tachycardia and a rise in respiratory rates. On the contrary, the use of clinical signs may lack accuracy in the evaluation of hypotension and require additional testing for guiding the management of PPH.

The findings revealed that blood loss is correlated with modifications in clinical signs, but it is complicated to establish robust cut-offs that can guide the management of women with pregnancy-associated hemorrhage. On the contrary, the shock index findings are more endorsing when it comes to a clinical sign derivative. The shock index is computed as the heart rate divided by the systolic blood pressure, and this simple computation may change unstable metrics into a more precise enabler of hypovolemia. The shock index may reveal hypovolemia even in patients who would be undertaken with no hypotension.^22,23^ In addition, the shock index has been recommended as an approach for predicting mortality because of hypovolemic shock in trauma patients. The use of the shock index is promising even in obstetric populations when identifying and evaluating early bleeding.^24^

Blood transfusion is usually required for the treatment of severe anemia and shock resulting from PPH. In developed countries, the easy accessibility and the safe use of blood components resulted in an abundant use of blood transfusion, specifically in the postpartum period. On the contrary, the existing awareness of transfusion-related complications and the cost triggered a reassessment of this transfusion practice. This study had limitations. The data set encompassed merely Indian women, and it is not clear whether the outcomes can be extrapolated to women of other ethnic groups. Furthermore, the data were collected from two hospitals. This study also did not include women with risk factors for PPH. The small sample size of the individuals with PPH in the study limits its generalizability to a larger population and its robustness. In addition, some of the listed clinical symptoms and signs of PPH occur in women without this ailment, which is indicative of a lack of specificity. Hence, PPH cannot be prevented by recognizing clinical signs and symptoms of blood loss, as by then hemorrhage has already occurred.

## Conclusions

The clinical findings, such as tachycardia, dizziness, and palpitation, were not found to be associated with blood loss since most of them were found in women with <500 mL of blood loss. Therefore, these cannot be used as indicators for the initiation of management of PPH in the absence of methods for assessing the exact amount of blood loss by drapes and changes in blood parameters. However, symptoms, such as sweating, weakness, hypotension, restlessness, and oliguria, can indicate PPH and can therefore be used to facilitate early treatment. During the antenatal and peripartum periods, extra vigilance is required for identifying women at risk and facilitating early intervention and treatment of PPH. As PPH can occur in women without any risk factors, there is a need for protocols in place for the management of PPH in all women giving birth, regardless of any predefined risk factors.

## Abbreviation Used

CS: Caesarean Section
Hb: Hemoglobin
PCV: Packed Cell Volume
PPH: Post Partum Hemorrhage
