# Supplementary material for: Determining the Correlation Between Blood Loss and Clinical Findings Among Patients with Postpartum Hemorrhage
Source: Womens Health Rep (New Rochelle). 2025 Jan 8;6(1):37–42. doi: 10.1089/whr.2024.0103 (PMC11773172; doi:10.1089/whr.2024.0103)
Supplement: Supplementary Data S1 [file whr.2024.0103_supp_datas1.docx]

**Study Novelty**

***What is already known about the study?***

- There is currently lack of studies correlating clinical factors due to blood loss caused by PPH and analyzing clinical presentation due to body’s compensatory mechanism to hypovolemia in PPH.
- It is important to record these clinical symptoms as they can be an indicator of hypovolemia in patients and a speedy intervention could be provided without any delay.

***Study Contribution/Value***

- The study identified the signs that will help the midwives or the healthcare providers attending deliveries to prevent patient from going into hypovolemic shock, especially when immediate testing is not possible.
- The clinical findings associated with the blood loss include tachycardia, dizziness, palpitation, sweating, weakness, hypotension, restlessness, oliguria, and confusion, which can be used for initiation of management of PPH in absence of methods for assessing the exact amount of blood loss by drapes and change in blood parameters.

***Practical Implications on the field of Study***

- Extra vigilance is needed in order to identify women at risk and facilitate early intervention for preventing post-partum hemorrhage.
- There is a need for protocols in place for the management of post-partum haemorrhage in all women giving birth, regardless of any predefined risk factors
